# Effect of the addition of sulfated polysaccharides of animal and algal origin in the freezing medium of *Colossoma macropomum* (Characiformes: Serrasalmidae)

**DOI:** 10.1590/1984-3143-AR2025-0105

**Published:** 2025-12-01

**Authors:** Carlos Henrique Sousa de Melo, Marcos Luiz da Silva Apoliano, Yara Silvino Sales, Renata Vieira do Nascimento, Vanessa Alves Pereira, Emanuel Martins da Costa, Jéssica Sales Lobato, José Ariévilo Gurgel Rodrigues, Carminda Sandra Brito Salmito-Vanderley

**Affiliations:** 1 Laboratório de Biotecnologia da Reprodução de Peixes, Programa de Pós-graduação em Ciências Veterinárias, Universidade Estadual do Ceará, Fortaleza, CE, Brasil; 2 Curso de Medicina Veterinária, Centro Universitário Inta, Umirim, CE, Brasil; 3 Engenharia de Pesca, Universidade Estadual Vale do Acaraú, Camocim, CE, Brasil; 4 Departamento de Engenharia de Pesca, Universidade Federal do Ceará, Fortaleza, CE, Brasil

**Keywords:** Tambaqui, semen freezing, cryoprotection, sperm kinetics

## Abstract

The aim of this study was to evaluate the effects of supplementing the cryodiluent medium with sulfated polysaccharides (SP) extracted from marine algae (*Ascophyllum nodosum* or *Solieria filiformis*) and fish skin (*Colossoma macropomum*, *Prochilodus brevis*, or *Oreochromis niloticus*) on the cryopreservation of tambaqui semen. Twenty male tambaqui were used for semen collection and cryopreservation. For the fertilization assay, three males and five females were used. In Experiment 1, different concentrations of SP (0.0, 0.1, 0.25, 0.5, and 0.75 mg/mL) extracted from fish skin or marine algae were added to the freezing medium for *C. macropomum* semen. In Experiment 2, the results of sperm velocity analyses were used to select one concentration of each sulfated polysaccharide for use in fertilization trials. Among the treatments, *A. nodosum* at 0.75 mg/mL and *C. macropomum* at 0.50 mg/mL stood out, significantly improving sperm parameters such as motility, VCL, VSL, VAP, and LIN compared to the control group. *S. filiformis*, *P. brevis*, and *O. niloticus* also showed good results, with performance varying by concentration. Membrane integrity was higher in the algae-derived extract groups. Sperm morphology and DNA integrity did not differ significantly among groups. Fertilization rates remained high across all treatments (84.67% to 88.67%), with no statistically significant differences, indicating that the tested extracts did not compromise fertility. It was concluded that supplementation with SP from *A. nodosum* at 0.75 mg/mL and *C. macropomum* at 0.50 mg/mL, although all treatments showed similar fertility rates, is recommended as an additive to the semen dilution medium for tambaqui during freezing, as it improved important sperm parameters such as motility and VCL.

## Introduction

The *Colossoma macropomum* (Characiformes: Serrasalmidae) is a native fish species from the Amazon and represents a promising alternative with enormous potential to be explored (Associação Brasileira da Piscicultura, 2025). Among the main native species, round fish stand out due to their strong performance, economic viability, hardiness, and sensory characteristics that appeal to consumers (Pires et al., 2017; Marcos et al., 2020; Freitas et al., 2021).

Global fisheries and aquaculture face challenges such as extreme climate events, pollution, and biodiversity loss, reinforcing the need for sustainable and efficient solutions (FAO, 2024). In response to these challenges, semen cryopreservation and assisted fertilization represent valuable tools that, when integrated into genetic improvement programs, can help reverse this scenario in a relatively short time. Moreover, these techniques offer several advantages, including the rational use of gametes, conservation of genetic material, exchange of semen between producers in different regions, and the preservation of genetic diversity within populations (Beirão et al., 2019).

However, one of the limitations of the cryopreservation technique is the occurrence of cellular damage during the process, which can compromise sperm quality after thawing. The increased production of reactive oxygen species (ROS) is one of the main factors responsible for these negative impacts on semen quality. Thus, to minimize the resulting damage, several antioxidants have been proposed and tested with the aim of optimizing sperm cryopreservation in fish (Alevra et al., 2022).

Sulfated polysaccharides (SP) are complex polymers found in macroalgae and animal cells and possess several well-known biological properties (Gupta and Abu-Ghannam, 2011; Jegadeshwari and Rajaram, 2024). SP extracted from the skin of *Oreochromis niloticus* have shown positive effects on the membrane integrity of tambaqui sperm when incorporated into cryopreservation media (Pereira et al., 2021). Furthermore, the addition of SP derived from the red macroalga *Solieria filiformis* to the cryodiluent contributed to improved preservation of sperm morphology in the same species (Pereira et al., 2020). The beneficial effects of macroalgae-derived SP supplementation in cryopreservation media have also been reported for *Prochilodus brevis* (Nascimento et al., 2021; Nascimento et al., 2022). The alteration of the SP used and its concentrations can modify its action.

Therefore, the aim of this study was to evaluate the effects of supplementing the cryodiluent medium with SP extracted from marine algae (*Ascophyllum nodosum* or *S. filiformis*) and fish skin (*C. macropomum*, *P. brevis*, or *O. niloticus*) on the cryopreservation of tambaqui semen.

## Methods

### Ethical aspects and experimental design

The project was approved by the Animal Ethics and Research Committee of the State University of Ceará (approval number 09279405/2021). The study was conducted at the Laboratory of Fish Reproduction Biotechnology (LBRP) (3°47'36.2”S; 38°33'30.1”W) of the State University of Ceará and at the Marine Biochemistry Laboratory (BIOMAR) of the Federal University of Ceará (UFC), in Fortaleza, Ceará, Brazil.

The project was divided into two experimental stages. In Experiment 1, different concentrations of SP (0.0 mg/mL, 0.1 mg/mL, 0.25 mg/mL, 0.5 mg/mL, and 0.75 mg/mL) extracted from fish skin (*C. macropomum*, *P. brevis*, or *O. niloticus*) or from marine algae (*A. nodosum* or *S. filiformis*) were added to the freezing medium of *C. macropomum* semen. In experiment 2, velocity analysis results were used to select one concentration of each sulfated polysaccharide for fertilization trials.

### Experimental animals and gamete collection

Twenty male tambaqui from the broodstock of the Laboratory of Fish Reproduction Biotechnology at the State University of Ceará were used, maintained at the Center for Biotechnology Applied to Aquaculture at the Federal University of Ceará. Fish with an average weight of 5 kg were selected, meeting the necessary criteria to indicate reproductive maturity (Pereira et al., 2020). Reproduction was induced in these animals through the intracoelomic application of 0.3 pellet/kg of Ovopel^®^.

After 14 hours, the animals were sedated in a Eugenol solution (Sigma-Aldrich^®^, San Luis, Missouri, USA) at a 1:10:10000 ratio (Eugenol: alcohol: water) until loss of equilibrium was observed in the fish. Subsequently, the animals were positioned in lateral recumbency, and a gentle craniocaudal abdominal massage was performed to collect the semen using sterile 3 mL syringes.

Immediately after collection, the samples were transferred to polyethylene tubes and kept in thermal boxes (4°C) during transport to the Laboratory of Fish Reproduction Biotechnology at the State University of Ceará. In the laboratory, the samples were analyzed, semen pools were formed, and subsequently cryopreserved.

### Cryopreserved sperm of C. macropomum

Semen samples with motility above 80%, after activation, were used to form pools (n = 8), with each pool containing semen from three animals (a total of 20 fish). The pools were diluted (1:9 semen:diluent) and frozen in a solution containing 5% glucose (Neon^®^, Suzano, São Paulo, Brazil) and 10% dimethyl sulfoxide (DMSO) (Neon^®^, Suzano, São Paulo, Brazil). In Experiment 1, the cryopreservation medium was supplemented with different concentrations of SP (0.1 mg/mL, 0.25 mg/mL, 0.5 mg/mL, and 0.75 mg/mL) of extracted from fish skin (*C. macropomum*, *P. brevis*, or *O. niloticus*) or from marine algae (*A. nodosum* or *S. filiformis*). Thus, a total of 20 treatments were established. A non-supplemented solution was used as a control.

The samples were packaged in 0.25 mL straws, sealed with polyvinyl alcohol, and left for 10 minutes at approximately 10°C to allow equilibration. The straws were then placed in a dry shipper, where they remained for 15 minutes to allow freezing in liquid nitrogen vapor (−170ºC). Immediately afterward, the samples were stored in a liquid nitrogen container (−196ºC). After 45 days, the samples were thawed by immersion in a water bath at 45ºC for 8 seconds. Subsequently, the sample was subjected to sperm evaluation.

### Sperm analysis

The integrity of the sperm membrane was assessed by staining with eosin-nigrosin. A smear of the pool was made, containing a final volume of 10 μL in a ratio of 1:2:2 (semen:eosin:nigrosin). Using an optical microscope (400×, Opton Microscope; Tucsen, China), 200 sperm cells per slide were analyzed. Unstained sperm cells were considered to have intact membranes, while sperm cells showing pink/red staining were considered to have ruptured membranes.

For sperm morphology analysis, the semen was fixed in a 4% formaldehyde citrate solution (10:100; semen:fixative) and then stained with Bengal Rose in a ratio of 1:10 (stain:fixed semen). Two slides per pool were prepared, and 100 sperm cells per slide were evaluated using an optical microscope (400×). Sperm cells were classified as normal when no morphological alteration was observed or abnormal when head or tail abnormalities were detected.

A computer-assisted sperm analysis (CASA) system was used to assess kinetic parameters using the Sperm Class Analyzer software (SCA, Microptics, Barcelona, Spain, version 3.2), which was configured for fish with 50 images per second. Aliquots of sperm solution (5 μL) were deposited in Makler chambers and activated with 100 μL of 125 mM NaCl. Kinetic analyses were performed approximately 15 seconds after sperm cell activation. The evaluated parameters were total motility (%), curvilinear velocity (VCL - μm/s), straight-line velocity (VSL - μm/s), average path velocity (VAP - μm/s), linearity (LIN - %), and straightness (STR -%). Each analysis assessed at least 1000 sperm cells.

DNA integrity analysis was based on the sperm chromatin fragmentation rate, conducted using the SCD (Sperm Chromatin Dispersion) test, according to the methodology of Almeida-Monteiro et al. (2020). Briefly, 1 μL of sperm solution was diluted in 1.5 mL of phosphate-buffered saline (PBS) and kept in a water bath at 37°C until use. Then, 25 μL of the PBS-sperm solution was mixed with 50 μL of low-molecular-weight agarose (Low-melt Agarose; Sigma-Aldrich^®^, San Luis, Missouri, USA). Aliquots of 2 μL of this mixture were deposited at each of the 10 points on a slide previously prepared with highly purified agarose (NA Agarose; Sigma-Aldrich^®^, San Luis, Missouri, USA). The slides were then covered with coverslips and placed on a chilled metal surface at 4°C for five minutes. Subsequently, the coverslips were removed, and the slides were subjected to different solutions: acid solution (hydrochloric acid (HCl) and distilled water, for seven minutes); lysis solution (sodium chloride (NaCl), sodium dodecyl sulfate (SDS), Triton X, ethylenediaminetetraacetic acid (EDTA), β-mercaptoethanol, Tris-HCl solution, and distilled water, for 25 minutes); distilled water (for five minutes), and 70% alcohol, 90% alcohol, and absolute alcohol, respectively (for two minutes each). After the bath, the samples were stained with the Panotic kit (RenyLab^®^, Condado de Barbacena, Minas Gerais, Brazil), with each slide being immersed in each stain for 10, 20, and 20 seconds, respectively. Finally, the slides were washed in distilled water and dried at room temperature. Two hundred sperm cells were evaluated using a phase contrast optical microscope coupled with a camera (200×; Nikon Eclipse 50i, Tokyo, Japan) to investigate the incidence of a halo around the sperm head. Cells with an external halo indicated sperm chromatin dispersion (intact DNA), while cells without a halo indicated DNA fragmentation.

### Fertilization

After conducting the experiment to determine the optimal concentration of each source of sulfated polysaccharides, fertilization tests and monitoring of embryo development were performed.

For this, *C. macropomum* broodstock that exhibited reproductive maturity indicators, such as semen release under slight abdominal pressure and hyperemic urogenital papilla for males, and a swollen abdomen and hyperemic urogenital papilla for females, were selected and induced to reproduce. The males (n=3) received a single dose of the synthetic hormone Ovopel^®^ at 0.3 pellet/kg of body weight, according to Souza et al. (2018), while the females (n=4; average weight of 7.8 ± 1.1 kg) received two doses of carp pituitary extract (CPE), both administered intracoelomically. The first dose for females was 0.5 mg of CPE/kg of body weight. After 12 hours of induction, the second CPE dose was administered to females at a concentration of 5.0 mg of CPE/kg of body weight (Woynarovich and Van-Anrooy, 2019), along with a single application of Ovopel^®^ to the males. The three males used in this phase belonged to the control group of 100% fresh semen, collected to form a pool at the time of fertilization, which was conducted simultaneously with the fertilization of previously cryopreserved semen samples.

After the second hormonal induction in females, the water temperature of the broodstock was monitored at one-hour intervals to calculate degree-hours, which consist of the sum of temperatures from the last induction, coinciding with the approximate time of final maturation and ovulation in females. For tambaqui, degree-hours range from 260 to 280 (Woynarovich and Van-Anrooy, 2019). The animals were then sedated in an Eugenol (Sigma-Aldrich^®^, San Luis, Missouri, USA) solution at a ratio of 1:10:10,000 (Eugenol: alcohol: water), and semen and oocytes were collected by gently massaging the abdomen in the craniocaudal direction. The samples were subsequently stored under refrigeration in graduated tubes and polyethylene containers, respectively.

Aliquots of the samples were analyzed for sperm and oocyte concentrations. Sperm concentration was calculated by counting cells in a 1.0 μL semen aliquot using a Neubauer chamber with the aid of an optical microscope (Opton Microscope; Tucsen, China) at 400× magnification. Oocyte concentration was determined by counting 1.0 g of the sample under a binocular stereomicroscope with 0.8 to 5× magnification (Opton, China).

Fertilization was performed using the dry method, in which sperm cells were carefully mixed with oocytes following the proportion recommended by Leite et al. (2013) of 5.0 × 10^4^ to 2.0 × 10^6^ sperm cells per oocyte. Then, 100 mL of water was added for hydration, followed by gentle homogenization for one minute. Subsequently, the fertilized eggs were transferred to cylindrical incubators in a water recirculation system, where they were maintained for embryonic development monitoring and fertilization rate assessment.

For the calculation of the fertilization rate, a sample of embryos was carefully collected from each incubator and placed in a Petri dish. Subsequently, the percentage of fertilized eggs was determined by counting 200 embryos under a stereomicroscope. The fertilization rate was assessed when the embryos reached the gastrula stage (Coelho et al., 2021).

### Statistics

The data were expressed as mean ± standard deviation. The Shapiro-Wilk test was applied to verify normality. Parametric results were subjected to analysis of variance (ANOVA), and then the means were compared using Tukey’s test. For non-parametric data, the Kruskal-Wallis test was applied to evaluate differences between groups. Finally, Dunn’s post-hoc test with Bonferroni correction was used for multiple comparisons between groups. Differences were considered significant when P < 0.05.

## Results

The semen collected from all animals was used to create eight pools, which showed an average total motility rate of 97.5 ± 3.2%, VCL of 80.2 ± 14.8 μm/s, VSL of 34.9 ± 10.4 μm/s, and VAP of 58.8 ± 16.8 μm/s. The proportion of morphologically normal spermatozoa was 84.50 ± 8.84%, and the values for plasma membrane integrity and sperm DNA integrity were 92.4 ± 1.7% and 93.0 ± 2.75%, respectively.

Regarding the percentage of motile spermatozoa (Table 1), the group *A. nodosum* at a concentration of 0.75 mg/mL (22.51 ± 1.69%), *C. macropomum* at concentrations of 0.25 mg/mL (21.33 ± 2.55%), 0.50 mg/mL (24.73 ± 3.30%), and 0.75 mg/mL (23.28 ± 2.65%), *P. brevis* at 0.10 mg/mL (23.28 ± 2.39%) and 0.50 mg/mL (21.76 ± 2.12%), and *O. niloticus* at 0.75 mg/mL (20.62 ± 0.77%) were statistically superior to the control group (17.13 ± 0.56%) (P<0.05). Within the experimental groups (Table 1), the concentrations of *A. nodosum* at 0.75 mg/mL, *C. macropomum* at 0.50 mg/mL and 0.75 mg/mL, and *P. brevis* at 0.10 mg/mL were statistically superior to *S. filiformis* at 0.10 mg/mL (18.68 ± 0.80%) and 0.25 mg/mL (17.90 ± 2.93%) (P<0.05).

**Table 1:** Effect of different extracts and concentrations on the sperm kinetic parameters (Motility, VCL, VSL, VAP, and LIN) of tambaqui.

	**Groups**	**Motility (%)**	**VCL (µm/s)**	**VSL (µm/s)**	**VAP (µm/s)**	**LIN (%)**
Control	0.00 mg/mL	17.13 ± 0.56	15.69 ± 0.40	1.59 ± 0.19	4.76 ± 0.22	10.12 ± 1.13
*A. nodosum*	0.10 mg/mL	20.11 ± 1.81^Bab^	17.74 ± 0.95*^Babc^	2.62 ± 0.46^*Bab^	6.45 ± 0.69^*Bab^	14.70 ± 2.09^*Aab^
	0.25 mg/mL	19.92 ± 0.68^Bab^	17.95 ± 1.49^*Babc^	2.58 ± 0.70^*Bab^	6.43 ± 1.08^*Bab^	14.21 ± 2.91^*Aab^
	0.50 mg/mL	20.09 ± 0.90^Bab^	18.13 ± 0.99^*Bab^	2.79 ± 0.57^*ABab^	6.17 ± 0.54^*Bab^	14.96 ± 2.18^*Aab^
	0.75 mg/mL	22.51 ± 1.69^*Aa^	19.89 ± 1.42^*Aa^	3.28 ± 0.63^*Aa^	7.33 ± 0.88^*Aa^	16.40 ± 2.16^*Aa^
*S. filiformis*	0.10 mg/mL	18.68 ± 0.80^Ab^	16.47 ± 0.77^Bbc^	1.81 ± 0.40^Bb^	5.19 ± 0.65^Bb^	10.93 ± 2.12^Bb^
	0.25 mg/mL	17.90 ± 2.93^Ab^	16.49 ± 0.78^ABbc^	2.17 ± 0.20^ABab^	5.77 ± 0.16^ABab^	13.12 ± 0.74^Aab^
	0.50 mg/mL	19.66 ± 1.73^Aab^	17.68 ± 1.47^*Aabc^	2.47 ± 0.70^Aab^	6.03 ± 0.65^Aab^	13.76 ± 2.67^Aab^
	0.75 mg/mL	19.13 ± 0.61^Aab^	17.57 ± 1.13^*ABabc^	2.45 ± 0.50^ABab^	6.13 ± 0.79^*Aab^	13.84 ± 2.16^Aab^
*C. macropomum*	0.10 mg/mL	19.82 ± 1.79^Cab^	16.45 ± 0.24^Abc^	1.97 ± 0.53^Ab^	5.51 ± 0.93^Ab^	11.50 ± 2.56^Ab^
	0.25 mg/mL	21.33 ± 2.55^*BCab^	16.94 ± 1.06 ^Aabc^	2.28 ± 0.49^Aab^	5.88 ± 0.71^Aab^	13.41 ± 2.45^Aab^
	0.50 mg/mL	24.73 ± 3.30^*Aa^	17.32 ± 0.88^Aabc^	2.24 ± 0.43^Aab^	5.94 ± 0.78^Aab^	12.85 ± 1.98^Aab^
	0.75 mg/mL	23.28 ± 2.65^*ABa^	16.67 ± 0.79^Aabc^	2.10 ± 0.26^Aab^	5.66 ± 0.41^Aab^	12.24 ± 0.57^Aab^
*P. brevis*	0.10 mg/mL	23.28 ± 2.39^*Aa^	16.50 ± 0.68^Abc^	1.89 ± 0.40^Bb^	5.49 ± 0.67^Ab^	11.41 ± 2.17^Bb^
	0.25 mg/mL	19.28 ± 1.88^Bab^	16.61 ± 0.70^Aabc^	2.04 ± 0.32^ABab^	5.66 ± 0.67^Aab^	12.22 ± 1.59^ABab^
	0.50 mg/mL	21.76 ± 2.12^*Aab^	16.57 ± 0.61^Aabc^	2.02 ± 0.35^ABab^	5.64 ± 0.42^Aab^	12.78 ± 2.50^ABab^
	0.75 mg/mL	19.23 ± 1.98^Bab^	16.86 ± 0.93^Aabc^	2.30 ± 0.39^Aab^	5.95 ± 0.60^Aab^	13.62 ± 2.03^Aab^
*O. niloticus*	0.10 mg/mL	20.28 ± 2.11^Aab^	15.92 ± 0.86^Bc^	1.76 ± 0.36^Bb^	5.10 ± 0.67^Bb^	10.97 ± 1.69^Cb^
	0.25 mg/mL	19.65 ± 1.43^Aab^	16.41 ± 0.95^ABbc^	1.97 ± 0.38^ABb^	5.47 ± 0.68^ABb^	11.97 ± 1.77^BCab^
	0.50 mg/mL	20.67 ± 2.64^Aab^	16.77 ± 0.41^Aabc^	2.15 ± 0.16^Aab^	5.77 ± 0.46^Aab^	12.81 ± 0.73^ABab^
	0.75 mg/mL	20.62 ± 0.77^*Aab^	16.71 ± 0.79^Aabc^	2.25 ± 0.42^Aab^	5.75 ± 0.61^Aab^	14.08 ± 1.62^*Aab^

Total motility; Curvilinear velocity – VCL; Straight line velocity – VSL; Average path velocity – VAP and Linearity – LIN. Values are expressed as mean ± standard deviation. An asterisk (*) indicates a statistically significant difference compared to the control group (p < 0.05). Different capital letters indicate statistically significant differences between the concentrations of sulfated polysaccharides within each group (p < 0.05). Different lowercase letters indicate statistically significant differences between concentrations of sulfated polysaccharides (p < 0.05).

Regarding VCL (Table 1), all concentrations of *A. nodosum*, as well as the 0.50 mg/mL and 0.75 mg/mL concentrations of *S. filiformis*, showed statistically higher values compared to the control group (P<0.05). The other treatments did not differ significantly from the control (Table 1). The 0.75 mg/mL concentration of *A. nodosum* showed better results (19.89 ± 1.42 μm/s), with statistically significant differences compared to *S. filiformis* at concentrations of 0.10 mg/mL (16.47 ± 0.77 μm/s) and 0.25 mg/mL (16.49 ± 0.78 μm/s), *C. macropomum* at 0.10 mg/mL (16.45 ± 0.24 μm/s), *P. brevis* at 0.10 mg/mL (16.5 ± 0.68 μm/s), and *O. niloticus* at 0.10 mg/mL (15.92 ± 0.86 μm/s) and 0.25 mg/mL (16.41 ± 0.95 μm/s) (Table 1) (P<0.05).

In terms of VSL (Table 1), all concentrations of *A. nodosum* were statistically superior to the control (P<0.05). The other treatments did not differ significantly from the control. The concentration of 0.75 mg/mL *A. nodosum* (3.28 ± 0.63 μm/s) (Table 1) showed better results and a statistically significant difference compared to *S. filiformis* at a concentration of 0.10 mg/mL (1.81 ± 0.40 μm/s), *C. macropomum* at 0.10 mg/mL (1.97 ± 0.53 μm/s), *P. brevis* at 0.10 mg/mL (1.89 ± 0.40 μm/s), and *O. niloticus* at both 0.10 mg/mL (1.76 ± 0.36 μm/s) and 0.25 mg/mL (1.97 ± 0.38 μm/s) (P<0,05).

The evaluation of the VAP variable revealed that all groups supplemented with *A. nodosum*, as well as the group supplemented with *S. filiformis* at a concentration of 0.75 mg/mL, showed values that were significantly higher than those of the control group (P<0.05) (Table 1). The concentration of 0.75 mg/mL *A. nodosum* (7.33 ± 0.88 μm/s) showed the highest VAP mean (Table 1) and was statistically superior to several groups, including *S. filiformis* at 0.10 mg/mL (5.19 ± 0.65 μm/s), *C. macropomum* at 0.10 mg/mL (5.51 ± 0.93 μm/s), *P. brevis* at 0.10 mg/mL (5.49 ± 0.67 μm/s), *O. niloticus* at both 0.10 mg/mL (5.10 ± 0.67 μm/s) and 0.25 mg/mL (5.47 ± 0.68 μm/s) (P<0.05).

For the LIN variable (Table 1), all groups supplemented with *A. nodosum*, as well as the group supplemented with *O. niloticus* at a concentration of 0.75 mg/mL, showed statistically higher values compared to the control group (P < 0.05). The group supplemented with *A. nodosum* at a concentration of 0.75 mg/mL (16.40 ± 2.16%) showed the highest mean for this parameter and was significantly superior to the groups *S. filiformis* at 0.10 mg/mL (10.93 ± 2.12%), *C. macropomum* at 0.10 mg/mL (11.50 ± 2.56%), *P. brevis* at 0.10 mg/mL (11.41 ± 2.17%), and *O. niloticus* at 0.10 mg/mL (10.97 ± 1.69%) (P < 0.05) (Table 1). None of the experimental groups showed a negative effect on the sperm kinetic variables.

The comparison of sperm kinetic parameters among the different concentrations of *A. nodosum* showed that the 0.75 mg/mL (Table 1) concentration resulted in significantly higher values compared to the other concentrations for the percentage of motile sperm (22.51 ± 1.69%), VCL (19.89 ± 1.42 µm/s), VSL (3.28 ± 0.63 µm/s), and VAP (7.33 ± 0.88 µm/s) (P < 0.05). For *S. filiformis* (Table 1), the 0.50 mg/mL concentration showed superior values compared to 0.10 mg/mL for VCL (17.68 ± 1.47 vs. 16.47 ± 0.77 µm/s) and VSL (2.47 ± 0.70 vs. 1.81 ± 0.40 µm/s). Regarding VAP and LIN, the concentrations of 0.50 mg/mL (6.03 ± 0.65 µm/s and 13.76 ± 2.67%) and 0.75 mg/mL (6.13 ± 0.79 µm/s and 13.84 ± 2.16%) performed better than the 0.10 mg/mL concentration (5.19 ± 0.65 µm/s and 10.93 ± 2.12%) (P < 0.05).

Within the *C. macropomum* groups (Table 1), the concentrations of 0.50 mg/mL (24.73 ± 3.30%) and 0.75 mg/mL (23.28 ± 2.65%) resulted in higher percentages of motile sperm compared to 0.10 mg/mL (19.82 ± 1.79%) (P < 0.05). In the *P. brevis* groups, the concentrations of 0.10 mg/mL (23.28 ± 2.39%) and 0.50 mg/mL (21.76 ± 2.12%) showed significantly higher motility percentages than the other concentrations (P < 0.05). Regarding the VSL and LIN parameters, the 0.75 mg/mL concentration (2.30 ± 0.39 µm/s and 13.62 ± 2.03%, respectively) was superior to 0.10 mg/mL (1.89 ± 0.40 µm/s and 11.41 ± 2.17%) in *P. brevis* (P < 0.05).

For *O. niloticus* (Table 1), comparison between its concentrations revealed that 0.50 mg/mL (16.77 ± 0.41; 2.15 ± 0.16; and 5.77 ± 0.46 µm/s for VCL, VSL, and VAP, respectively) and 0.75 mg/mL (16.71 ± 0.79; 2.25 ± 0.42; and 5.75 ± 0.61 µm/s) were significantly superior to 0.10 mg/mL (15.92 ± 0.86; 1.76 ± 0.36; and 5.10 ± 0.67 µm/s) (P < 0.05). For the LIN parameter, only the 0.75 mg/mL concentration (14.08 ± 1.62%) outperformed 0.10 mg/mL (10.97 ± 1.69%) (P < 0.05).

Regarding membrane integrity (Table 2), the control group was considered a lost replicate for this parameter, and therefore, statistical comparisons with the control group were not possible. The addition of SP extracted from marine macroalgae, at all tested concentrations of *A. nodosum* and at 0.10 mg/mL, 0.25 mg/mL, and 0.50 mg/mL of *S. filiformis*, resulted in better membrane integrity outcomes compared to animal-derived (P < 0.05). None of the experimental groups had a negative effect on this variable.

**Table 2:** Sperm parameters (% membrane integrity, % normal morphology, and % fragmented DNA) in different experimental groups treated with *A. nodosum*, *S. filiformis*, *C. macropomum*, *P. brevis*, and *O. niloticus*, compared to the control.

	**Groups**	**Membrane integrity (%)**	**Normal morphology (%)**	**DNA integrity (%)**
Control	0.00 mg/mL	-	56.88 ± 5.67	80.0 ± 5.96
*A. nodosum*	0.10 mg/mL	57.70 ± 13.12^ABab^	59.31 ± 5.25^Aab^	86.75 ± 4.27^ABa^
	0.25 mg/mL	69.50 ± 5.59^Aa^	55.56 ± 5.83^ABab^	84.75 ± 8.15^ABa^
	0.50 mg/mL	43.07 ± 5.00^Babc^	51.50 ± 6.12^Bab^	87.86 ± 3.36^Aa^
	0.75 mg/mL	45.00 ± 4.45^Babc^	53.88 ± 8.11^ABab^	80.50 ± 7.67^Ba^
*S. filiformis*	0.10 mg/mL	42.62 ± 7.61^Aabcd^	49.94 ± 5.00^Cb^	78.71 ± 11.29^Aa^
	0.25 mg/mL	43.86 ± 9.58^Aabcd^	60.69 ± 7.08^Aa^	81.31 ± 5.61^Aa^
	0.50 mg/mL	42.79 ± 5.41^Aabcd^	52.19 ± 7.35^BCab^	79.00 ± 7.45^Aa^
	0.75 mg/mL	27.64 ± 4.31^Babcd^	56.62 ± 5.48^ABa^	82.44 ± 4.62^Aa^
*C. macropomum*	0.10 mg/mL	22.75 ± 4.87^ABbcd^	56.19 ± 4.96^ABab^	78.75 ± 6.50^Aa^
	0.25 mg/mL	23.00 ± 3.73^Abcd^	58.81 ± 5.42^Aab^	81.81 ± 6.45^Aa^
	0.50 mg/mL	20.09 ± 3.00^ABbcd^	56.94 ± 4.84^ABab^	81.19 ± 5.15^Aa^
	0.75 mg/mL	18.03 ± 1.51^Bc^	53.36 ± 2.34^Bab^	82.38 ± 4.58^Aa^
*P. brevis*	0.10 mg/mL	19.01 ± 3.56^Bcd^	56.50 ± 7.21^Aab^	82.25 ± 6.34^Aa^
	0.25 mg/mL	21.05 ± 3.64^ABbcd^	58.19 ± 6.43^Aab^	82.25 ± 6.09^Aa^
	0.50 mg/mL	24.05 ± 3.04^Aabcd^	60.56 ± 6.04^Aab^	83.62 ± 6.07^Aa^
	0.75 mg/mL	18.02 ± 2.77^Bc^	55.31 ± 5.98^Aab^	80.56 ± 5.14^Aa^
*O. niloticus*	0.10 mg/mL	28.21 ± 9.16^Aabcd^	56.94 ± 6.47^Aab^	87.79 ± 2.36^Aa^
	0.25 mg/mL	24.71 ± 6.49^ABabcd^	56.75 ± 5.61^Aab^	80.00 ± 5.35^Ba^
	0.50 mg/mL	27.50 ± 5.41^Aabcd^	57.31 ± 4.49^Aab^	82.19 ± 4.66^Ba^
	0.75 mg/mL	19.07 ± 5.13^Babcd^	56.62 ± 4.90^Aab^	82.62 ± 5.28^Ba^

Values are expressed as mean ± standard deviation. Different capital letters indicate statistically significant differences between the concentrations of sulfated polysaccharides and the control (p < 0.05). Different lowercase letters indicate statistically significant differences between concentrations of sulfated polysaccharides (p < 0.05).

For normal morphology (Table 2), no significant differences were observed between the treatments supplemented with SP extracted from either animal or plant sources and the control (P > 0.05). In the group supplemented with *S. filiformis*, the concentration of 0.25 mg/mL (60.69 ± 7.08%) showed a higher average of sperm with normal morphology compared to the 0.10 mg/mL (49.94 ± 5.00%) concentration (P < 0.05). None of the experimental groups had a negative effect on this variable.

Normal morphology parameters (Table 2) varied among the tested concentrations of *A. nodosum* and demonstrated that the 0.10 mg/mL concentration (59.31 ± 5.25%) was superior to the 0.50 mg/mL group (51.50 ± 6.12%) (P < 0.05). For the *S. filiformis* groups, the 0.25 mg/mL concentration (60.69 ± 7.08%) was significantly higher than both 0.10 mg/mL (49.94 ± 5.00%) and 0.50 mg/mL (52.19 ± 7.35%) (P < 0.05). In the treatments with *C. macropomum*, the 0.25 mg/mL group (58.81 ± 5.42%) showed superior results compared to the 0.75 mg/mL group (53.36 ± 2.34%) (P < 0.05). No significant differences were observed among the concentrations tested in the groups supplemented with *P. brevis* and *O. niloticus* (Table 2).

DNA integrity did not differ significantly between treatments with sulfated polysaccharides of either animal or plant origin and the control group (Table 2) (P > 0.05). None of the experimental groups exhibited a negative impact on this parameter. However, when comparing the different concentrations of *A. nodosum*-derived polysaccharides, the 0.50 mg/mL concentration (87.86 ± 3.36%) was significantly higher than the 0.75 mg/mL concentration (80.50 ± 7.67%) (Table 2) (P < 0.05). In the groups treated with *O. niloticus*-derived polysaccharides (Table 2), the 0.10 mg/mL concentration (87.79 ± 2.36%) demonstrated superior results compared to the other tested concentrations of the same origin (P < 0.05).

**Figure 1:**
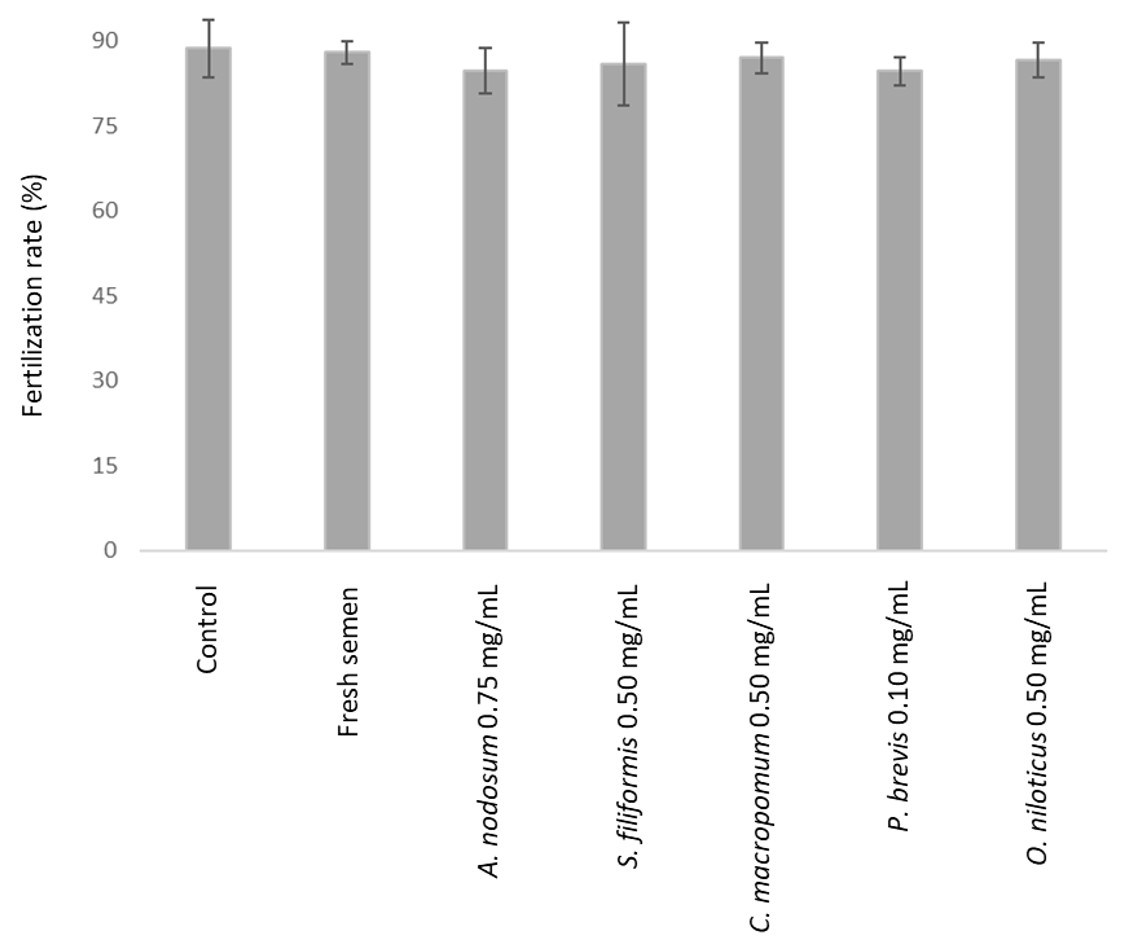
Effect of different treatments on fertilization rate (%) of tambaqui.

For the evaluation of fertility, different treatments and concentrations were selected to assess their potential effects (Figure 1). The control group presented a fertility rate of 88.67 ± 5.03%, while the group using fresh semen showed a similar performance, with 88.00 ± 2.00%. Among the treatments with algae or fish extracts, *C. macropomum* at 0.50 mg/mL demonstrated a mean fertility of 87.00 ± 2.65%, followed by *O. niloticus* at 0.50 mg/mL with 86.67 ± 3.06%, *S. filiformis* at 0.50 mg/mL with 85.87 ± 7.37%, *A. nodosum* at 0.75 mg/mL with 84.81 ± 4.03% and *P. brevis* at 0.10 mg/mL with 84.67 ± 2.52%. Not possible to detect a statistically significant difference between the groups regarding fertilization (P>0,05).

## Discussion

The present research investigated the efficacy of two sources of sulfated polysaccharides: algal (*A. nodosum* or *S. filiformis*) and animal (*C. macropomum*, *P. brevis*, or *O. niloticus*). This study is the first report on the use of sulfated polysaccharides extracted from *A. nodosum*, *C. macropomum* and *P. brevis* in the cryopreservation of tambaqui semen. It was observed that supplementation of the extender with different sources of sulfated polysaccharides had a beneficial effect on membrane integrity, percentage of motile sperm, and sperm kinetics, with these parameters differing significantly from the control group.

Sperm kinetic parameters are important indicators for evaluating fish semen and are associated with high fertilization rates. Among these parameters, motility and VCL stand out for their greater importance and should be considered key markers of semen quality (Viveiros et al., 2010; Cydzik et al., 2025). In the present study, variations in the type and concentration of sulfated polysaccharides had distinct effects on sperm kinetic parameters, which may suggest differential affinities between the polysaccharides and tambaqui semen.

There is no universally optimal cryodiluent for different fish species, and both the specific characteristics of the supplement and its concentration are important factors in improving outcomes. The effects of adding supplements to culture media or cryoprotectants are cell-type dependent and influenced by both the physicochemical properties of the supplement and its concentration (Cabrita et al., 2011; O'Neill et al., 2022).

A previous study (Pereira et al., 2021) had determined that the use of 5% glucose, combined with lower concentrations of sulfated polysaccharides, is safer and more effective in maintaining and preserving tambaqui sperm cells. Thus, the present study compared different sources of sulfated polysaccharides at low concentrations, and fertilization trials were performed using the best kinetic result from each tested source.

In this study, the treatment supplemented with 0.75 mg/mL of *A. nodosum* (Table 1) stood out compared to the control group and several other treatments in terms of sperm motility percentage (22.51 ± 1.69%), VCL (19.89 ± 1.42 µm/s), VSL (3.28 ± 0.63 µm/s), VAP (7.33 ± 0.88 µm/s) and LIN (16.40 ± 2.16%). Overall, the data suggest that supplementation with SP derived from algae was more effective than those of animal origin. Notably, the *A. nodosum* groups showed particularly promising results.

*A. nodosum* is a species predominantly found along the rocky shores of the North Atlantic, particularly in cold-water regions. Rayirath et al. (2009) demonstrated that lipophilic compounds extracted from *A. nodosum* significantly enhance freezing tolerance in *Arabidopsis thaliana*. Furthermore, Nair et al. (2012) confirmed that the lipophilic fraction of *A. nodosum* promotes the accumulation of osmoprotectants, and metabolomic analysis revealed increased sugar content and altered fatty acid unsaturation factors that contribute to membrane stabilization under low-temperature conditions. Our study suggests that the sulfated polysaccharide extract from *A. nodosum* may exert a similar protective effect during semen cryopreservation, contributing to improved post-thaw sperm quality.

For the animal-derived SP in this study, the concentration of 0.50 mg/mL from *C. macropomum* (Table 1) stood out from the others, particularly for differing from the control in the percentage of motile spermatozoa (24.73 ± 3.30%). Variations in the effect of SP addition to the cryopreservation medium are expected, as the chemical structures of sulfated polysaccharides are complex and may vary depending on their origin. The degree of sulfation is fundamental to the antioxidant activity of SP due to their electron-donating potential, which enables them to neutralize free radicals. Thus, SP may exert different effects depending on their degree of interaction with biomolecules and cells (Pereira et al., 2021; Nagahawatta et al., 2023).

The preservation of membrane integrity was one of the parameters positively influenced by the addition of SP to the extender (Table 2). Fish semen is particularly prone to oxidative damage due to the high content of polyunsaturated fatty acids present in their membranes (Félix et al., 2021). Membrane integrity is an important factor to ensure sperm cell survival and motility for the period required to achieve fertilization (Cosson, 2004). The group treated with *A. nodosum* showed high membrane integrity values (43%–60%) with no significant differences among the concentrations. The group treated with 0.25 mg/mL of *A. nodosum* presented the highest value (69.50 ± 5.59) in the Table 2, suggesting that this extract may effectively preserve or protect sperm membrane integrity, even at higher concentrations. The positive effect of low concentrations of *O. niloticus* supplementation on membrane integrity had already been reported by Pereira et al. (2020).

In general, SP of animal origin showed inferior performance compared to those of algal origin with respect to membrane integrity (Table 2). One of the main distinguishing factors of marine algal SP is their high content and strategic positioning of sulfate groups, which confer distinct structural properties and enable specific binding to membrane-associated proteins and extracellular matrix components. This interaction can modulate biological processes such as cell adhesion, proliferation, and stabilization of membrane integrity (Nagahawatta et al., 2023; Jegadeshwari and Rajaram, 2024).

In this study, supplementation of the cryodiluent medium with algal- or animal-derived SP did not significantly affect sperm morphology compared to the control (Table 2). However, for the supplementation with *S. filiformis*, a statistical difference was observed between the 0.10 mg/mL and 0.25 mg/mL groups, suggesting a moderate dose-dependent positive effect, with a significant improvement in morphology at a specific concentration. These results are consistent with findings from previous studies, indicating that SP supplementation does not exert toxic effects on sperm cells (Pereira et al., 2021; Nascimento et al., 2022).

Regarding the percentage of intact DNA (Table 2), no statistical difference was observed between the treatments and the control. In fish species with external fertilization, fertilization depends primarily on the presence of numerous spermatozoa near the egg and on a greater ability to with stand hyposmotic stress when released into the water (Herrera et al., 2021). This characteristic may facilitate and compensate for the presence of some sperm carrying DNA damage.

In general, an intact DNA percentage of approximately 90% in fish sperm is considered ideal for maintaining high fertilization potential. However, this threshold has been reported for only a limited number of species (Pérez-Cerezales et al., 2010) and has not yet been specifically established for *Colossoma macropomum*. Therefore, in the present study, this value is used as a comparative reference based on related teleost species, rather than as a species-specific standard.. It is also reasonable to consider that the capacity for DNA repair after fertilization may vary among species, being a parameter still poorly understood for tambaqui, which could exert a compensatory effect in relation to the percentage of damage in sperm DNA.Although we observed that the parameters evaluated in this study were influenced by the origin and concentration of the SP, no statistical difference was found between treatments in the fertilization assay (Figure 1). This result may be associated with the high sperm-to-egg ratio used in the fertilization trials, which ranged from 5.0 × 10^4^ to 2.0 × 10^6^ sperm cells per oocyte.

## Conclusion

In conclusion, it is suggested that supplementation with SP from *A. nodosum* at 0.75 mg/mL and *C. macropomum* at 0.50 mg/mL, although all treatments showed similar fertility rates, is recommended as an additive to the semen dilution medium for tambaqui during freezing, as it improved important sperm parameters such as motility and VCL.
